# To Our Contributors

**Published:** 1892-06

**Authors:** 


### TO OUR CONTRIBUTORS.

We must urge again the rule of this journal, that all contributions or correspondence intended for the Editorial Department must be *sent direct to the Editor, No.* 3243 *Chestnut Street, Philadelphia.* Further, matter intended for publication in any given month, such as meeting notices, proceedings of societies, must be in his hands not later than the 5th.

Two rules should ever be present in the minds of writers,—first, to take the utmost care in the preparation of a paper; and, secondly, to write it so that it will give the least trouble to those who are obliged to arrange it for publication. The following suggestions may be of some value:

Write on one side of the sheet, beginning the first page half way down. Allow a margin of a half inch on the left side. Separate the lines, allowing space for the editor to make necessary corrections. Write in a round, clear hand, or have the paper type-written. Condense as much as possible.

Very few, we imagine, have any idea of the time wasted by editors, compositors, and proof-readers by the continued neglect of these simple but important hints.

